# Complete Genome Sequence of the Cluster P Mycobacteriophage Phegasus

**DOI:** 10.1128/mra.00540-22

**Published:** 2022-08-04

**Authors:** Abigail A. Howell, Cyril J. Versoza, Gabriella Cerna, Tyler Johnston, Shriya Kakde, Keith Karuku, Maria Kowal, Jasmine Monahan, Jillian Murray, Teresa Nguyen, Aurely Sanchez Carreon, Elizabeth Song, Abigail Streiff, Blake Su, Faith Youkhana, Saige Munig, Zeel Patel, Minerva So, Makena Sy, Sarah Weiss, Yang Zhou, Susanne P. Pfeifer

**Affiliations:** a School of Life Sciences, Arizona State University, Tempe, Arizona, USA; b Biodesign Institute, Arizona State University, Tempe, Arizona, USA; c Center for Evolution and Medicine, Arizona State University, Tempe, Arizona, USA; d School of Molecular Sciences, Arizona State University, Tempe, Arizona, USA; e Division of Biology and Medicine, Brown University, Providence, Rhode Island, USA; f School of Politics and Global Studies, Arizona State University, Tempe, Arizona, USA; g Center for Mechanisms of Evolution, Arizona State University, Tempe, Arizona, USA; Portland State University

## Abstract

We characterized the complete genome of the cluster P mycobacteriophage Phegasus. Its 47.5-kb genome contains 81 protein-coding genes, 36 of which could be assigned a putative function. Phegasus is most closely related to two subcluster P1 bacteriophages, Mangethe and Majeke, with an average nucleotide identity of 99.63% each.

## ANNOUNCEMENT

A diverse range of bacteriophages is known to infect Mycobacterium smegmatis (1). As part of the Howard Hughes Medical Institute Science Education Alliance—Phage Hunters Advancing Genomics and Evolutionary Science (HHMI SEA-PHAGES) program, we characterized the complete genome of Phegasus, a putatively temperate cluster P, subcluster P1 mycobacteriophage.

Phegasus was obtained from a soil sample collected from the manure area of a horse barn at the Guilford Riding School (Guilford, CT; 41.3029 N, 72.6537 W) through enriched isolation, purification, and amplification in Mycobacterium smegmatis mc^2^155, following the procedures outlined in the SEA-PHAGES Discovery Guide (https://seaphagesphagediscoveryguide.helpdocsonline.com/home). A dual-indexed sequencing library was prepared from genomic DNA using the NEBNext Ultra II FS kit and sequenced on an Illumina MiSeq instrument (coverage: >900×). Following Russell (2), Newbler v.2.9 was used to *de novo* assemble the 307,831 single-end (150-bp) reads into a full-length genome sequence, with a 12-base 3′ sticky overhang. The 47,578-bp genome exhibits a GC content of 67.4%. The completeness, accuracy, and genomic termini were checked using Consed v.29.0 (3). All software was executed using default settings.

**FIG 1:**
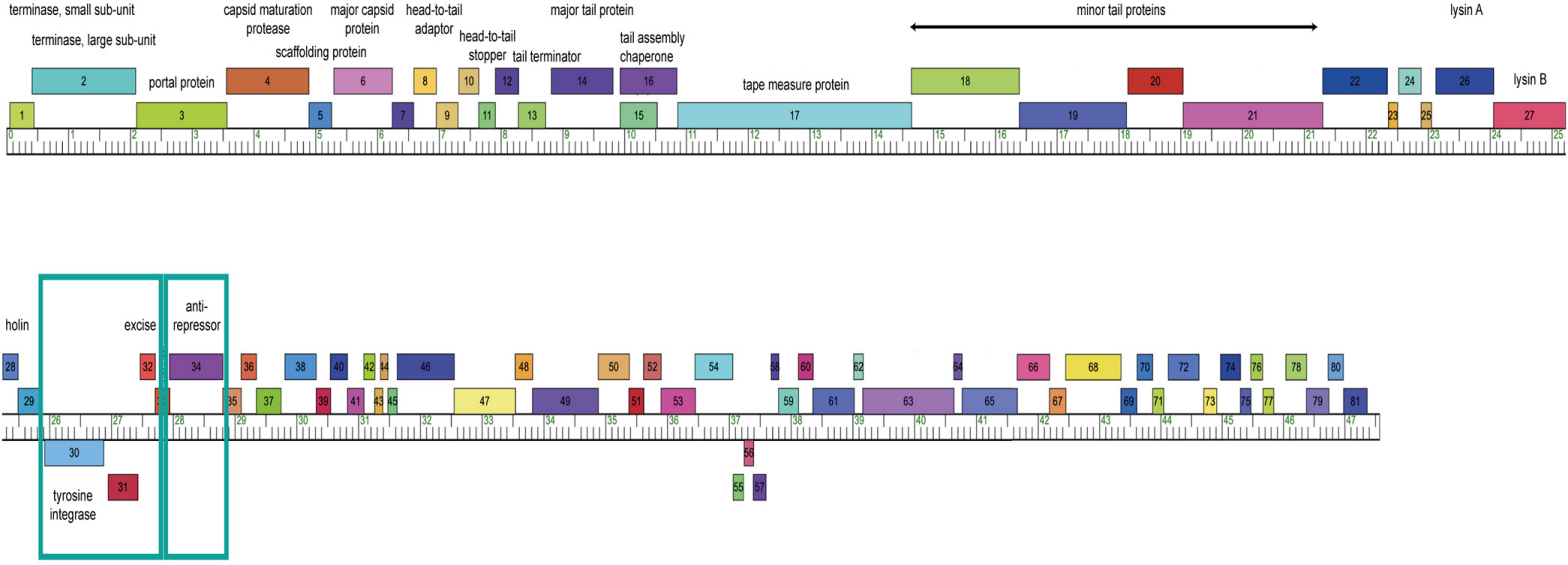
Genome of the cluster P mycobacteriophage Phegasus. Protein-coding genes on the forward or reverse strands with their putative functional assignments (if available) are displayed above or below the ruler, respectively. The integration-dependent immunity system (genes 30 to 32 and 34) is indicated by teal-colored boxes. ssDNA, single-stranded DNA.

Genome annotation followed the HHMI SEA-PHAGES Bioinformatics Guide (https://seaphagesbioinformatics.helpdocsonline.com/home), using GLIMMER v.3.0.2 (4) and GeneMark v.2.5 (5) embedded within DNA Master v.5.23.6 to identify open reading frames. Eighty-one protein-coding genes were predicted in the genome (gene density: 1.70 genes/kb), of which 36 could be assigned a putative function using NCBI BLAST (6) and HHpred (7), as well as information on synteny obtained using Phamerator (8). Of the remaining genes, five were classified as membrane proteins using TMHMM v.2.0 (9) and SOSUI v.1.11 (10). The left arm of the genome encodes several well-conserved structural and assembly proteins (including small and large terminase subunits, a portal protein, capsid maturation protease, a scaffolding protein, a major capsid protein, both a head-to-tail adapter and stopper, a tail terminator, a major tail protein, two tail assembly chaperones, a tape measure protein, and four minor tail proteins). Following the structural proteins is a lysin cassette, comprised of lysin A and lysin B, responsible for the cleavage of the host cell wall during the final stages of the lytic cycle. The right arm of the genome encodes nonstructural genes, including an integration-dependent immunity system (genes 30 to 32 and 34) that governs the transition from the lysogenic to lytic state (Fig. 1). A partial tRNA (located at positions 26972 to 27076) was identified using tRNAscan-SE v.2.0 (Infernal score, 12.6) (11), which may represent either the remnants of a full-length tRNA or part of a tRNA that is assembled after integration into the host genome.

Multiple sequence alignments were generated using MAFFT v.7 (12), which demonstrated that Phegasus is most closely related to two subcluster P1 bacteriophages, Mangethe (GenBank accession number MK016499) and Majeke (MF472894), collected at the University of KwaZulu-Natal in South Africa, with an average nucleotide identity of 99.63% each.

### Data availability.

The whole-genome sequencing data are available at NCBI’s Sequence Read Archive (accession number SRR19912416 and BioProject accession number PRJNA488469). The annotated genome assembly is available at NCBI GenBank under accession number ON637760.
